# Identification of Predictive Biomarkers Based on Cytokine Profiles for Molecular Relapse After Treatment-Free Remission in Chronic Myeloid Leukemia Patients

**DOI:** 10.3390/cells15090791

**Published:** 2026-04-27

**Authors:** Bianca Vasconcelos Cordoba, Carolina Pavlovsky, María Belén Sanchez, Elena Beatriz Moiraghi, Ana Ines Varela, Miguel Arturo Pavlovsky, Isabel Amanda Giere, Franco Federico Freilich, Isolda Fernandez, Maria José Mela Osório, Ricardo Khalil Tannuri, Federico Sackmann, Mariana Juni, Georgina Emilia Bendek Del Prete, Eduardo Oscar Bullorsky, Juan Dupont, Josefina Freitas, Verónica Ventriglia, Ana Garcia Labanca, Romina Mariano, Carina Gumpel, Etelvina Macchiavello, Pedro Negri Aranguren, Mariano Paoletti, Francisca Rojas, Maria Fernanda Tosin, Marta Catalan, Maria del Rosario Custidiano, Maria Cecilia Foncuberta, Julio Cesar Sánchez Ávalos, Silvia Miranda, José Mordoh, Estrella Mariel Levy, Michele Bianchini

**Affiliations:** 1 Centro de Investigaciones Oncológicas–Fundación Cáncer (CIO-FUCA), Buenos Aires 1426, Argentina; 9mariabelensanchez@gmail.com (M.B.S.); josemordoh39@gmail.com (J.M.); estrellamlevy@yahoo.com.ar (E.M.L.); mbianchini@conicet.gov.ar (M.B.); 2 Fundación para Combatir la Leucemia (FUNDALEU), Buenos Aires 1114, Argentina; cpavlovsky@fundaleu.org.ar (C.P.); mpavlovsky@fundaleu.org.ar (M.A.P.); igiere@fundaleu.org.ar (I.A.G.); investigacion@fundaleu.org.ar (F.F.F.); ifernandez@fundaleu.org.ar (I.F.); mjmela@fundaleu.org.ar (M.J.M.O.); ktannuri@fundaleu.org.ar (R.K.T.); fsackmann@fundaleu.org.ar (F.S.); mjuni@fundaleu.org.ar (M.J.); 3 Hospital José María Ramos Mejía, Buenos Aires 1221, Argentina; beatrizmoiraghi@hotmail.com (E.B.M.); anainesvarelap@gmail.com (A.I.V.); 4 Hospital Italiano, Buenos Aires 1199, Argentina; gbendek89@gmail.com; 5 Hospital Británico, Buenos Aires 1280, Argentina; eduardo.bullorsky@hotmail.com; 6 Centro de Educación Médica e Investigaciones Clínicas Norberto Quirno (CEMIC), Buenos Aires 1425, Argentina; jcdupont643@gmail.com; 7 Hospital Posadas, Buenos Aires 1684, Argentina; mjosefinafreitas@hotmail.com (J.F.); veroventriglia@hotmail.com (V.V.); 8 Hospital Italiano, Mendoza 5519, Argentina; agdelabanca@hotmail.com; 9 Hospital San Martín, Parana 3100, Argentina; romimariano@hotmail.com; 10 Hospital Privado de Rosario, Santa Fe 2013, Argentina; carinagumpel@yahoo.com.ar; 11 Instituto Hematológico del Noroeste de Buenos Aires (IHNOBA), Buenos Aires 1663, Argentina; etelmac@hotmail.com; 12 Instituto Privado de Hematología y Hemoterapia, Parana 3100, Argentina; pedro.negri.aranguren@gmail.com; 13 Clínica 25 de Mayo, Mar del Plata 7600, Argentina; drmmdq@yahoo.com.ar; 14 Hospital de Clínicas José de San Martín, Buenos Aires 1120, Argentina; francarojas@hotmail.com; 15 Hospital El Cruce, Buenos Aires 5401, Argentina; fernanda.tosin@yahoo.com; 16 Clínica Monte Grande, Buenos Aires 1842, Argentina; martacatalan2002@yahoo.com.ar; 17 Instituto Alexander Fleming (IAF), Buenos Aires 1426, Argentina; rosario.custidiano@gmail.com (M.d.R.C.); cecilia.foncuberta@gmail.com (M.C.F.); jcsanchezavalos@yahoo.com.ar (J.C.S.Á.); 18 Instituto Alberto C. Taquini de Investigaciones en Medicina Traslacional (IATIMET), Buenos Aires 1122, Argentina; simir2@gmail.com

**Keywords:** chronic myeloid leukemia, treatment-free remission, cytokine profiling, immune biomarkers, MCP-1, IL-6

## Abstract

**Highlights:**

**What are the main findings?**

- In CML patients undergoing TKI discontinuation, longer treatment duration and deeper molecular responses were associated with improved molecular relapse-free survival.
- Baseline levels of IL-6 and MCP-1 identified patients at higher risk of relapse in a decision tree model and remained informative in an independent validation cohort.

**What are the implications of the main findings?**

- Lower circulating levels of IL-6 and MCP-1 were linked to increased relapse risk, suggesting a possible role of immune context in maintaining treatment-free remission.
- Cytokine profiling may complement molecular monitoring and contribute to a more refined risk assessment when considering TKI discontinuation.

**Abstract:**

Tyrosine kinase inhibitors (TKIs) have transformed chronic myeloid leukemia (CML) into a manageable disease, and discontinuation has become a feasible goal for many patients. However, molecular relapse after TKI cessation remains a challenge. This study investigated factors influencing molecular relapse-free survival (MRFS) in a real-world cohort of CML patients undergoing TKI discontinuation, focusing on clinical, molecular, and immune-related variables. Traditional prognostic tools, such as the Sokal score, showed limited capacity to predict relapse risk in this context. Instead, total treatment duration and depth of molecular response emerged as critical predictors, with longer therapy and deeper remission associated with improved outcomes. A key novel finding was the prognostic relevance of cytokine profiles, particularly interleukin-6 (IL-6) and monocyte chemoattractant protein-1 (MCP-1). These cytokines were strong predictors of relapse in a decision tree model that achieved high specificity and positive predictive value in an independent validation cohort. Notably, lower IL-6 and MCP-1 levels were associated with increased relapse risk, suggesting reduced immune surveillance may contribute to recurrence. Integrating cytokine signatures with molecular markers improved prognostic accuracy, supporting immune biomarkers as complements to established clinical parameters. These findings underscore the complexity of relapse mechanisms and the need for individualized risk assessment when considering TKI discontinuation.

## 1. Introduction

The introduction of tyrosine kinase inhibitors (TKIs) revolutionized the management of chronic myeloid leukemia (CML), shifting it from a fatal disease to a chronic, manageable condition with survival rates nearing those of the general population. Imatinib, the first TKI approved for CML treatment, demonstrated a 10-year overall survival rate of approximately 83.3%, marking a significant milestone in hematological oncology [1].

Despite this remarkable progress, the necessity for continuous, often lifelong, therapy presents multiple challenges, including drug-related adverse effects, patient adherence issues, considerable financial costs, and the potential for resistance development [2]. In this scenario, treatment-free remission (TFR) has emerged as an important and feasible therapeutic objective for selected patients. Clinical trials such as the TWISTER study have provided compelling evidence that a significant subset of patients who achieve a sustained deep molecular response (DMR) can successfully discontinue TKI therapy without experiencing molecular relapse [3]. Long-term follow-up data from several cohorts suggest that roughly 35 to 65% of patients maintaining stable DMR can preserve remission after stopping treatment [4]. Based on these findings, TFR is currently recommended after at least a minimum of three years of therapy, with at least two years in MR4.0 or deeper molecular remission [5,6]. However, persistent leukemic stem cells have been detected even in patients with long-term undetectable *BCR::ABL1* transcripts, suggesting that molecular remission alone may underestimate the true disease burden [7]. This underscores the need for additional biomarkers that more accurately capture residual disease and relapse risk, allowing a better prediction of which patients will maintain TFR.

Immune system dynamics have been increasingly implicated as key determinants of TFR success. The recovery and functional activity of immune effector cells, particularly natural killer (NK) cells, have been correlated with favorable outcomes post-TKI discontinuation. Specifically, higher frequencies of mature NK cell subsets have been associated with prolonged TFR, indicating an essential role of innate immunity in controlling residual leukemic cells [8,9,10]. Moreover, the broader immunological milieu, including T-cell subsets and cytokine profiles, appears to influence whether patients sustain remission or relapse after stopping treatment [11].

In this context, cytokines represent critical mediators of leukemic-immune interactions and have been proposed as candidate biomarkers for predicting TFR outcomes. Certain pro-inflammatory cytokines, such as IL-6 and MCP-1, have demonstrated associations with both molecular relapse and remission, depending on the clinical context and timing of measurement [12]. Conversely, interleukin-10 (IL-10) acts as a potent immunoregulatory cytokine by suppressing the activation of myeloid cells and limiting pro-inflammatory signaling pathways, thereby contributing to the fine-tuning of immune responses [13]. The dualistic role of cytokines reflects the complex interplay between inflammation, immune regulation, and leukemic biology in the context of TFR. Given this complexity, comprehensive cytokine profiling may improve our understanding of the immunological factors underlying TFR and aid in developing predictive models that transcend molecular monitoring alone.

While our previous study provided initial evidence supporting the prognostic value of low MCP-1 and IL-6 levels at the time of discontinuation, the analysis was restricted to the first Argentina Stop Trial cohort (AST-I), the initial cytokine panel and a shorter follow-up period [12]. Since then, several patients who initially maintained remission but later relapsed, and additional cytokines relevant to the leukemic–immune interface became available, making it possible to reassess these preliminary findings with greater statistical power. Building upon these developments, the present study incorporates extended follow-up of AST-I, a broader cytokine panel and an independent validation cohort to provide a more comprehensive framework for biomarker discovery and refinement.

In our real-world study, we aimed to identify plasma cytokine signatures that distinguish CML patients maintaining molecular remission following TKI discontinuation from those who relapse. Utilizing multiplex cytokine analysis, we compared the profiles of patients who experienced molecular relapse with those who maintained remission, to explore their potential as predictive biomarkers. Our findings contribute to refining the immunological landscape of TFR and may support the implementation of personalized strategies for TKI discontinuation, ultimately improving patient outcomes and quality of life.

## 2. Materials and Methods

### 2.1. Patient Samples

This study included patients with CML attempting TKI discontinuation. Peripheral blood samples were collected prior to discontinuation, following the inclusion criteria established for the enrollment of patients in the AST [14], which required patients to have maintained a DMR (*BCR::ABL1* ≤ 0.01%, or undetectable *BCR::ABL1* with at least 10,000 ABL1 transcripts, i.e., MR4.0 or better) for a minimum of two years (Table S1).

Patient recruitment, using the same inclusion criteria, was carried out in two stages: AST-I, which included 46 patients enrolled between February 2019 and March 2020, while AST-II included 35 patients enrolled between July 2022 and April 2023. The AST-I cohort was initially used for exploratory cytokine profiling using a broader panel, after which selected cytokines were measured in additional patients from the AST-II cohort. For all subsequent analyses, both cohorts were considered as a single dataset, and a seed-based cross-validation approach was applied to define training and validation subsets.

Patients were classified into two groups based on their molecular response after discontinuation: the Relapsed (R) group, defined by the loss of Major Molecular Response (MMR, *BCR::ABL1* > 0.1%), and the Non-Relapsed (NR) group, comprising those who maintained molecular remission. Patients in the R group restarted treatment with the same TKI, showing a good response. There was no progression to advanced stages or deaths associated with the disease.

### 2.2. Multiplex Plasma Cytokine Analysis by Luminex Technology

Peripheral blood samples anticoagulated with EDTA were collected before TKI stopping. First, blood was centrifuged at 400 *g* for 30 min at 4 °C. The upper phase was further centrifuged at 3800 *g* for 10 min and plasma samples were kept at −80 °C until use. No protease inhibitors were added, following the manufacturer’s recommendations for multiplex cytokine assays. The levels of 31 analytes, including cytokines, chemokines, growth factors (C, CK, and GF, respectively), and soluble transmembrane proteins, were measured in duplicate plasma samples using five different multiplex magnetic bead assays (Merck Millipore, Burlington, MA, USA) on a Magpix^®^ equipment (Merck-Millipore, Burlington, MA, USA). The measured analytes were: Eotaxin/CCL11, GM-CSF, G-CSF, IFNa2, IL-1a, IL-1b, IL-1Ra, IL-2, IL-3, IL-4, IL-6, IL-7, IL-8/CXCL8, IL-9, IL-10, IL-12(p70), IL-15, MCP-1/CCL2, MIP-1α/CCL3, TNFα, TGF-α, VEGF-A, LIF, SCF, TGF-β1, TGF-β2, TGF-β3, CD86/B7-2, PD-1, PD-L1, and PD-L2. Assays were performed following the manufacturers’ instructions. Standard curves and samples were tested in duplicate. Standards were plotted and concentrations were determined using xPONENT software (version 4.2).

### 2.3. Statistical Analysis

Statistical analyses were performed in GraphPad Prism (GraphPad Software Version 9.4.1). Non-parametric tests were used for quantitative variables due to their significant deviation from normality. The Mann–Whitney U test was employed to compare numeric variables between the R and NR groups, while the Chi-square test was used for categorical variables. MRFS rates were estimated using Kaplan–Meier analysis, and group comparisons were performed with the log-rank test. Quantitative variables were dichotomized based on Receiver Operating Characteristic (ROC) curves or medians, and survival curves were compared using the log-rank test. A *p*-value of less than 0.05 was considered statistically significant.

To preselect the most relevant variables for the predictive models, a non-parametric Spearman correlation analysis was performed between quantitative and qualitative variables, with variables being removed when their correlation exceeded 0.85. A 10-fold cross-validation approach, using a seed-based method to split the data into discovery and validation cohorts, was employed. A classification tree algorithm was applied to the variables selected after preprocessing. Models were chosen based on a difference of less than 0.01 between the discovery and validation sets in terms of the Area Under the Curve (AUC), ensuring model stability and external validity. All analyses were conducted using R statistical software (version 4.3.1), with the Caret package (version 6.0-94), pROC package (version 1.18.4), and rpart package (version 4.1.23).

As a final step, univariate Cox proportional hazards models were first used to evaluate the individual effect of each variable on relapse risk. An exploratory multivariable Cox model was then constructed to assess whether these associations, particularly for cytokines, remained consistent after adjustment for clinical variables. Given the number of events, subsequent analyses focused on parsimonious models to obtain more stable and interpretable estimates. Model performance was evaluated using Harrell’s concordance index (C-index). The C-index was calculated to assess prognostic discrimination.

## 3. Results

### 3.1. Patient Characteristics and TFR Outcomes

A total of 81 patients were included in the study, as shown in Table 1, which summarizes baseline characteristics and treatment details. Baseline clinical characteristics between AST-I and AST-II cohorts were compared to assess their comparability prior to integration into a single dataset. No significant differences in baseline clinical characteristics were observed between cohorts (Table S2).

**Figure 1:**
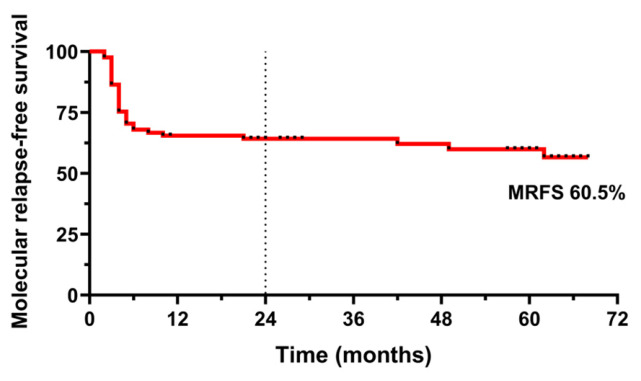
Molecular relapse-free survival after TKI discontinuation (*n* = 81). The solid red line represents the Kaplan–Meier estimate of survival. The dashed line indicates the 24-month survival probability. Dots correspond to censored cases.

At the time of the analysis, the median molecular follow-up after TKI discontinuation was 60 months (range, 20 to 69 months). Thirty-two patients (39.5%) lost MMR, most frequently within the first 6 months after TKI discontinuation (26 out of 32 patients), leading to a molecular relapse-free survival of 62% at 24 months and 60.5% for the entire follow-up period (Figure 1). Among the remaining relapses, six occurred beyond 6 months, including four late relapses after 21 months.

**Figure 2:**
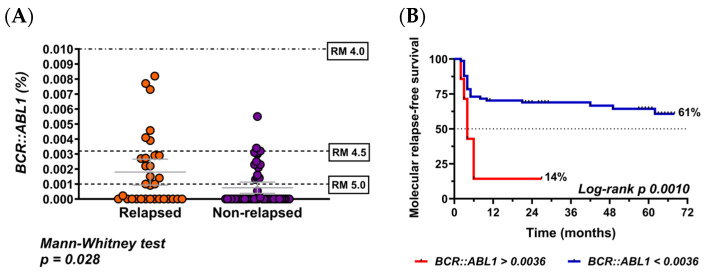

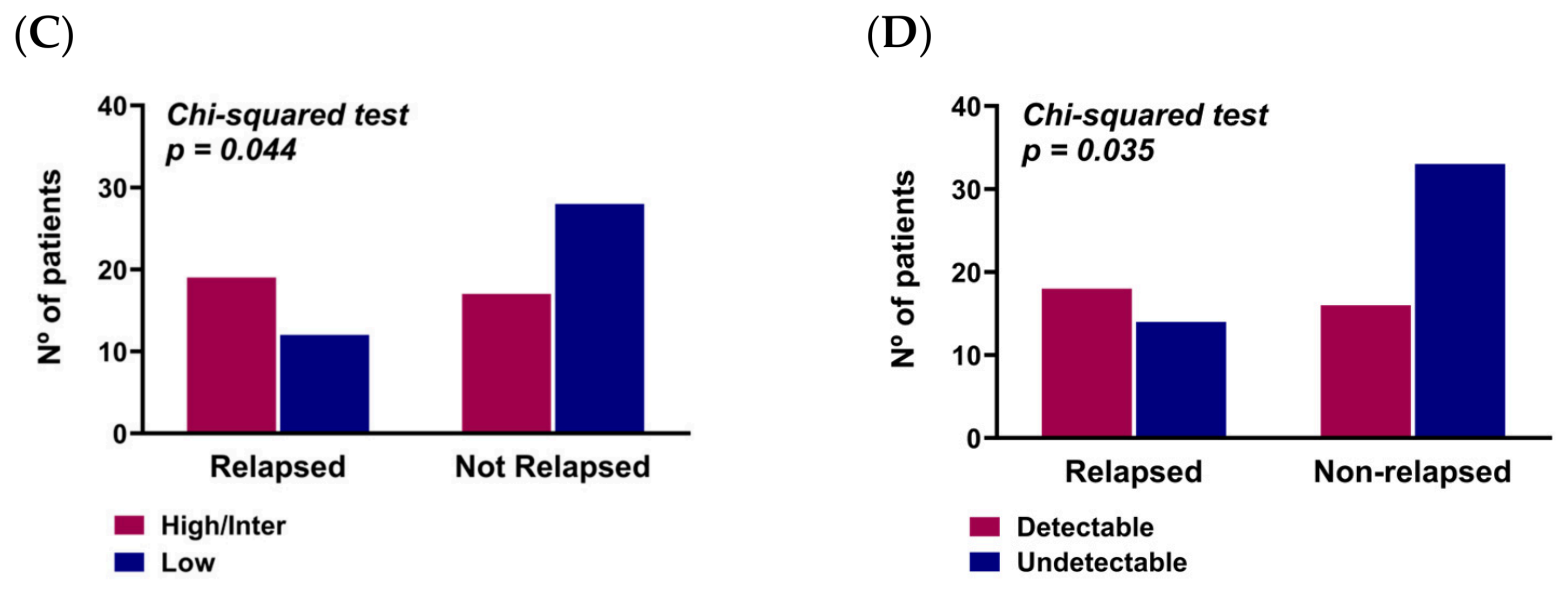
Treatment-free remission according to several variables of clinical relevance. (**A**) Comparison of the *BCR::ABL1* (%) levels between relapsed vs. non-relapsed patients. Colored dots represent individual patient values, the gray horizontal line indicates the median and the error bars represent data dispersion. (**B**) Molecular relapse-free survival according to *BCR::ABL1* (%) level (<0.0036% vs. >0.0036%). (**C**) Comparison of Sokal score between relapsed vs. non-relapsed patients. (**D**) Comparison of undetectable vs. detectable *BCR::ABL1* between relapsed vs. non-relapsed patients.

Associations between TFR and clinically relevant variables (age at diagnosis, age at enrollment, sex, treatment duration, *BCR::ABL1* detectability at enrollment, duration of DMR until discontinuation, time between treatment start and first DMR, TKI type prior to stop, *BCR::ABL1* (%) and Sokal score were evaluated to characterize the clinical context of the cohort. As expected, *BCR::ABL1* (%) was associated with TFR (Mann–Whitney test; *p* = 0.028) (Figure 2A). Using ROC curve analysis, the optimal cut off for *BCR::ABL1* (%) levels was determined to be 0.0036%, very close to the international standardized limit established for MR4.5. When patients were categorized based on this threshold, a significant difference in molecular relapse-free survival time was observed, with survival rates of 61% in the low group (<0.0036%) compared to 14% in the high group (≥0.0036%) (Log-rank test; *p* = 0.0010) (Figure 2B). Additionally, significant differences were observed between the groups with respect the Sokal score and *BCR::ABL1* detectability. A higher proportion of patients with a low Sokal score or undetectable *BCR::ABL1* was present in the NR group compared to the R group (chi-square test; *p* = 0.044 and *p* = 0.035, respectively). (Figure 2C,D).

**Figure 3:**
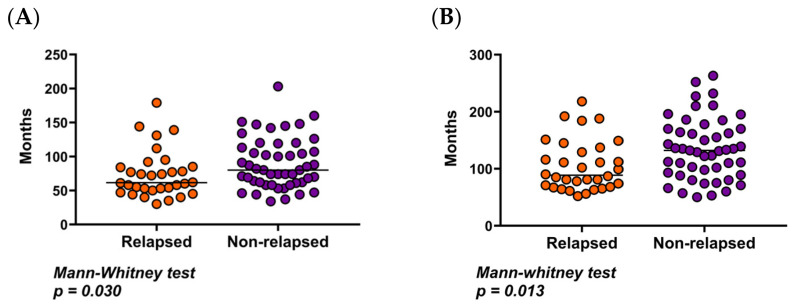
(**A**) Duration in months of DMR before stopping TKI in relapsed vs. non-relapsed patients. (**B**) Comparison of Treatment Duration in months between relapsed and non-relapsed Patients. Colored dots represent individual patient values and the black horizontal line indicates the median.

Significant differences were observed between NR and R patients in both the duration of DMR until discontinuation and the overall treatment duration (Mann–Whitney test; *p* = 0.030 and *p* = 0.013, respectively) (Figure 3A,B). The median duration of DMR was 80 months (range: 34–203) for NR patients and 61 months (range: 30–179) for R patients. Regarding total treatment duration, NR patients had a median of 132 months (range: 50–263), compared to 88 months (range: 52–218) in the R group.

It should be noted that, in our study, DMR duration was defined as the time from first achievement of DMR to TKI discontinuation, without confirmation of sustained DMR throughout this period, as confirmation was only systematically available during the two years immediately preceding therapy cessation.

### 3.2. Plasma Cytokines Profile at Baseline

Plasma levels of various cytokines, chemokines, and growth factors were assessed in CML patients at the time of treatment discontinuation, comparing those with and without molecular relapse. Of the 31 cytokines analyzed, 28 were detectable (IL-3, LIF, and TGFβ3 were below the limit of detection). Analysis of the AST-I cohort (Table 2) revealed that 4 cytokines exhibited significant differences. IL-12(p70) was significantly lower in non-relapsed patients (Mann–Whitney test; *p* = 0.007). In contrast, IL-6, MCP-1, and IL-8 levels were elevated in non-relapsed patients (Mann–Whitney test; *p* = 0.015; *p* = 0.005; *p* = 0.029, respectively).

In order to enlarge the analysis including new cytokines and more patients, we developed a new panel, by incorporating 7 additional analytes, in addition to IL-6 and MCP-1, that we decided to include again in the second cohorts of patients. Both cohorts were then analyzed together as a single dataset (Table 3).

**Figure 4:**
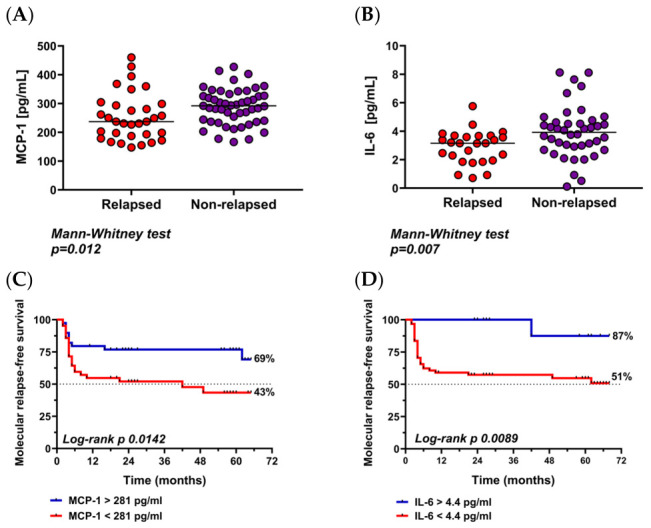
(**A**) Comparison of MCP-1 levels (pg/mL) in relapsed and non-relapsed groups. (**B**) Comparison of IL-6 levels (pg/mL) in relapsed and non-relapsed groups. Colored dots represent individual patient values, and the black horizontal line indicates the median. (**C**) Molecular relapse-free survival according to MCP-1 levels (<281 pg/mL vs. >281 pg/mL) at the time of discontinuation. (**D**) Molecular relapse-free survival according to IL-6 levels (<4.4 pg/mL vs. >4.4 pg/mL) at the time of discontinuation.

In the combined dataset, MCP-1 and IL-6 confirmed the pattern observed in AST-I; remaining significantly higher in the NR group, with median concentrations of 289.1 vs. 253.5 pg/mL (Mann–Whitney test; *p* = 0.012) and 3.9 vs. 2.9 pg/mL (Mann–Whitney test; *p* = 0.007), respectively (Figure 4A,B). Additionally, the optimal cutoffs for cytokine levels, determined based on ROC curve analysis, were 281 pg/mL for MCP-1 and 4.4 pg/mL for IL-6. When patients were categorized by these thresholds, a significant difference in molecular relapse-free survival was observed between the high and low groups, with 69% vs. 43% for MCP-1 (Log-rank test; *p* = 0.0142) and 87% vs. 51% for IL-6 (Log-rank test; *p* = 0.0089) (Figure 4C,D). However, no statistically significant differences were observed for other markers, including G-CSF, IL-10, IL-12 (p70), MIP-1a, TNF-α, and VEGFA (*p*-values ranging from 0.229 to 0.706).

### 3.3. Predictive Model for Discontinuation

To predict molecular relapse following TKI discontinuation, a decision tree algorithm was employed using data from the AST-I and AST-II cohorts. Two predictive models were developed: (1) a clinical model incorporating variables such as *BCR::ABL1* (%), *BCR::ABL1* detectability at enrollment, sex, age at diagnosis, treatment duration, duration of DMR until discontinuation, Sokal score, TKI type prior to stop and time to first DMR from treatment start; and (2) a cytokine model including G-CSF, IL-3, IL-10, TNF-α, VEGF-A, MCP-1, IL-6, and MIP-1α levels.

The cytokine model demonstrated superior predictive power compared to the clinical model, achieving an AUC of 0.7643 in the discovery cohort and 0.7708 in the validation cohort, whereas the clinical model achieved AUCs of 0.7063 and 0.6969, respectively. Accuracy, specificity, and positive predictive value were also higher for the cytokine model across cohorts, with an accuracy of 80% and a specificity of 100% in the validation cohort. In terms of sensitivity, the cytokine model reached 69.57% in the discovery cohort and 50% in the validation cohort. Despite lower sensitivity in the validation set, the overall improvement in predictive performance was statistically significant (*p* = 0.0102 and *p* = 0.0509 for the discovery and validation cohorts, respectively). The predictive performance of each model was assessed in discovery and validation cohorts (Table 4). A third model combining clinical and cytokine variables was also developed; however, this approach did not further enhance predictive performance compared to the cytokine model alone.

**Figure 5:**
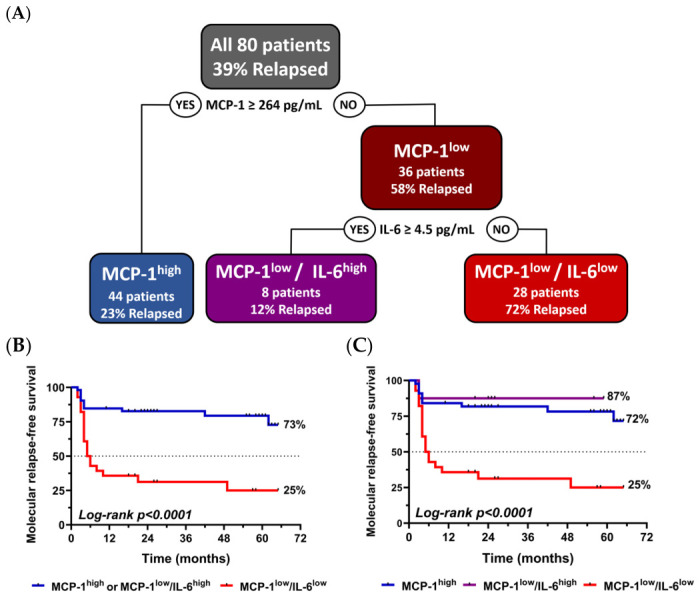
(**A**) Decision tree analysis for classification of patients according to molecular relapse status during treatment-free remission. (**B**) Molecular relapse-free survival for the MCP-1^high^ and MCP-1^low^/IL-6^high^ or MCP-1^low^/IL-6^low^ groups at the time of discontinuation. (**C**) Molecular relapse-free survival for the MCP-1^high^, MCP-1^low^/IL-6^high^ and MCP-1^low^/IL-6^low^ groups at the time of discontinuation.

Based on these results, a decision tree was generated to stratify patients into risk categories according to MCP-1 and IL-6 levels (Figure 5A). This tree included 80 patients (one extreme outlier was excluded to prevent distortion of MCP-1 and IL-6 threshold determination), with a relapse rate of 39%, defining stratification thresholds at MCP-1 ≥ 264 pg/mL and IL-6 ≥ 4.5 pg/mL.

The first split in the tree was based on MCP-1 levels. Patients with MCP-1 ≥ 264 pg/mL (MCP-1^high^) were at lower risk of relapse, with only 23% of the 44 patients experiencing molecular relapse. Conversely, patients with MCP-1 < 264 pg/mL (MCP-1^low^) were at higher risk, with 58% of the 36 patients relapsing.

Within the MCP-1^low^ group, IL-6 levels provided additional stratification. Patients with IL-6 ≥ 4.5 pg/mL (MCP-1^low^/IL-6^high^) showed the lowest relapse rate (12%, *n* = 8), while those with IL-6 < 4.5 pg/mL (MCP-1^low^/IL-6^low^) had the highest relapse rate, with 72% of the 28 patients in this subgroup experiencing molecular relapse.

Kaplan–Meier survival analysis was performed to evaluate molecular relapse-free survival across these cytokine-defined subgroups. In the first Kaplan–Meier curve (Figure 5B), patients were stratified into two groups: those with MCP-1^high^ or MCP-1^low^/IL-6^high^ and those with MCP-1^low^/IL-6^low^ levels. Patients in the MCP-1^high^ or MCP-1^low^/IL-6^high^ groups exhibited significantly longer relapse-free survival compared to the combined MCP-1^low^/IL-6^low^ subgroup (Log-rank test: *p* < 0.001).

In the second Kaplan–Meier analysis (Figure 5C), patients were categorized into three distinct groups: MCP-1^high^, MCP-1^low^/IL-6^high^ and MCP-1^low^/IL-6^low^. This refined stratification demonstrated that MCP-1^low^/IL-6^low^ patients had the shortest relapse-free survival, whereas MCP-1^high^ and MCP-1^low^/IL-6^high^ patients exhibited similarly favorable prognoses, further supporting the role of IL-6 in modulating relapse risk within the MCP-1^low^ group (Log-rank test: *p* < 0.001).

The performance of the cytokine model in relapse prediction was evaluated through confusion matrix analysis in both the discovery and validation cohorts (Table 5). In the discovery cohort, the model showed good sensitivity with 30 non-relapsed patients correctly identified as non-relapsed (true negatives) and 16 relapsed patients correctly classified as relapsed (true positives). However, there were 7 false negatives and 7 false positives. In the validation cohort, the model correctly identified 12 non-relapsed patients (true negatives) and 4 relapsed patients (true positives), with 4 false negatives and no false positives. These results highlight the model’s ability to predict relapse, although some misclassifications were observed.

### 3.4. Cox Regression Model

A univariate Cox regression analysis was performed to evaluate the individual effect of each variable on the risk of molecular relapse. Significant predictors included MCP-1 (HR 3.45, *p* = 0.002), *BCR::ABL1* > 0.0036% (HR 4.23, *p* = 0.002), treatment duration <111 months (HR 2.36, *p* = 0.026), and *BCR::ABL1* detectability at enrollment (HR 2.19, *p* = 0.031). IL-6, although showing a trend toward significance (HR 3.13, *p* = 0.060), did not reach statistical significance. Other variables such as IL-3, IL-10, and sex did not significantly influence the risk of relapse (*p* > 0.05). In addition to hazard ratios, model discrimination was assessed using Harrell’s concordance index (C-index). Univariate C-index values ranged from 0.58 to 0.64 across individual predictors, indicating moderate discriminatory ability. Among them, MCP-1, *BCR::ABL1* (%), and treatment duration achieved the highest concordance values, consistent with their strong hazard ratios and statistical significance (Table 6).

An exploratory multivariate Cox regression analysis was also conducted to assess the independent effects of clinical and molecular variables on relapse risk. In this model, MCP-1 levels below 264 pg/mL (HR = 2.94, 95% CI = 1.28–7.11, *p* = 0.012) and *BCR::ABL1* (%) levels above 0.0036% (HR = 3.62, 95% CI = 1.01–12.65, *p* = 0.042) remained independently associated with an increased risk of molecular relapse. The overall multivariate model demonstrated good prognostic discrimination, with a Harrell’s C-index of 0.77 (95% CI 0.69–0.85), supporting the model’s internal consistency and its ability to distinguish between patients at higher versus lower relapse risk (Table 7). Although its interpretation should be considered in light of the number of events and variables included.

To evaluate the behaviour of the cytokine pattern originally described in our earlier work, we also tested MCP-1^low^/IL-6^low^ as a combined variable in a multivariable Cox model using the same covariates as in Table 7. This combined cytokine pattern remained significantly associated with molecular relapse (HR = 3.47, 95% CI = 1.55–8.08, *p* = 0.003) and achieved a discriminative performance comparable to the model including each cytokine separately (Harrell’s C-index = 0.77, 95% CI 0.68–0.85). These results are presented in Table 8.

While the multivariable model provided an initial overview of the joint behavior of variables, bivariate and trivariate models were subsequently constructed to obtain more stable and interpretable estimates. These simpler models allowed for a more interpretable assessment of the individual contributions of MCP-1 and clinical parameters such as *BCR::ABL1* (%) and treatment duration, confirming their strong associations with relapse risk. Across all models, MCP-1 levels below 264 pg/mL were consistently associated with higher relapse risk, reinforcing its role as a key prognostic marker. *BCR::ABL1* (%) levels above 0.0036% also emerged as significant predictors, particularly in Models (2), (4), and (5). Treatment duration was also relevant in Models (3), (4), and (5), indicating that shorter treatment courses are linked to higher relapse probability. The inclusion of the C-index in these models enabled a direct comparison of their prognostic performance. Discrimination values between 0.68 and 0.72 confirmed the stability of the prognostic signal across different model configurations, supporting the robustness of the cytokine-based prognostic framework (Table 9).

**Figure 6:**
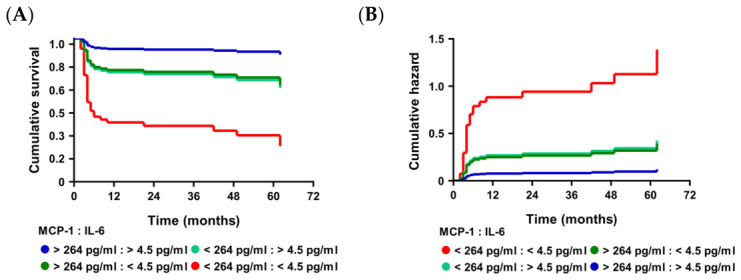

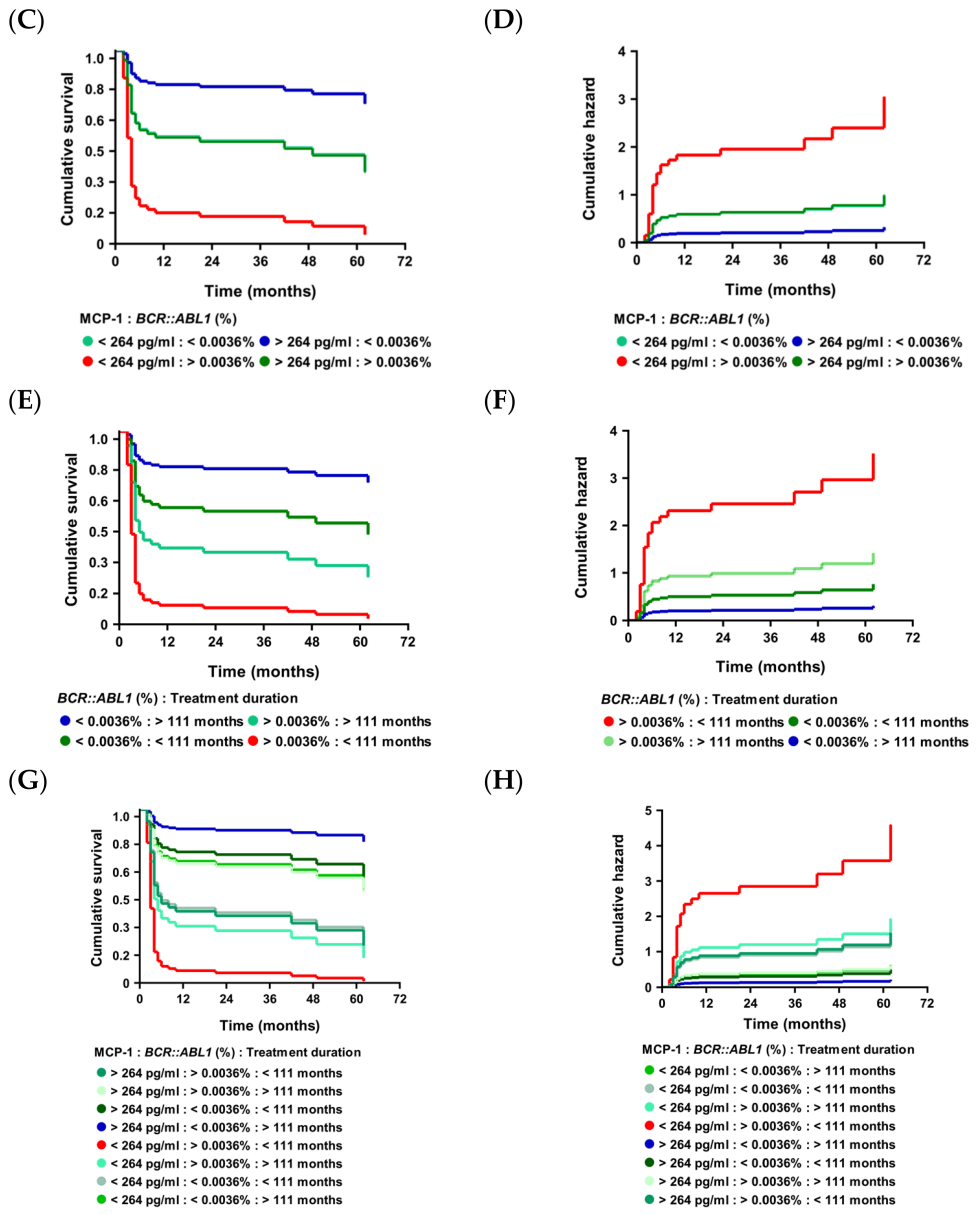
(**A**) Multivariate cumulative survival curve for MCP-1 and IL-6 levels estimated by the Cox regression model. (**B**) Multivariate cumulative hazard curve for MCP-1 and IL-6 levels estimated by the Cox regression model. (**C**) Multivariate cumulative survival curve for MCP-1 and *BCR::ABL1* (%) levels estimated by the Cox regression model. (**D**) Multivariate cumulative hazard curve for MCP-1 and *BCR::ABL1* (%) levels estimated by the Cox regression model. (**E**) Multivariate cumulative survival curve for MCP-1 and Treatment Duration estimated by the Cox regression model. (**F**) Multivariate cumulative hazard curve for MCP-1 and Treatment Duration estimated by the Cox regression model. (**G**) Multivariate cumulative survival curve for MCP-1, *BCR::ABL1* (%) levels and Treatment Duration estimated by the Cox regression model. (**H**) Multivariate cumulative hazard curve for MCP-1, *BCR::ABL1* (%) levels and Treatment Duration estimated by the Cox regression model.

Cumulative survival and hazard analyses were performed to assess MRFS across key clinical and molecular subgroups, as shown in Figure 6. These analyses were based on Cox regression models incorporating cytokine levels and clinical variables.

In the first survival analysis (Figure 6A), patients were stratified by MCP-1 and IL-6 levels. MCP-1^low^/IL-6^low^ patients showed a significantly higher risk of molecular relapse compared to MCP-1^high^ individuals (MCP-1 < 264 pg/mL; HR = 3.54, 95% CI = 1.68–8.00, *p* = 0.001), highlighting the role of MCP-1 and IL-6 in relapse prediction. The cumulative hazard curve (Figure 6B) supported this, showing a higher cumulative relapse risk in the MCP-1^low^/IL-6^low^ group.

The second model (Figure 6C) added *BCR::ABL1* (%) to MCP-1, revealing that patients with MCP-1^low^/*BCR::ABL1* > 0.0036% had the highest relapse probability, while MCP-1^high^/BCR::ABL1 < 0.0036% had the best relapse-free survival (MCP-1 < 264 pg/mL; HR = 3.06, 95% CI = 1.42–7.01, *p* = 0.006; *BCR::ABL1* > 0.0036%; HR = 3.10, 95% CI = 1.15–7.46, *p* = 0.018). The cumulative hazard analysis (Figure 6D) confirmed that MCP-1^low^/*BCR::ABL1* > 0.0036% patients had the highest cumulative relapse hazard.

In the third model (Figure 6E), focusing on clinical variables, *BCR::ABL1* (%) and treatment duration were stratified. Patients with *BCR::ABL1* > 0.0036%/treatment duration < 111 months had the highest relapse risk, while *BCR::ABL1* < 0.0036%/treatment duration > 111 months had the most favorable outcomes (*BCR::ABL1* > 0.0036%; HR = 4.59, 95% CI = 1.67–10.86, *p* = 0.001; treatment duration < 111 months; HR = 2.49, 95% CI = 1.19–5.53, *p* = 0.018). The cumulative hazard analysis (Figure 6F) corroborated these results.

The final model (Figure 6G) combined MCP-1 with clinical variables—treatment duration and *BCR::ABL1* (%). Patients with MCP-1^low^/*BCR::ABL1* > 0.0036%/treatment duration < 111 months showed the highest relapse risk (MCP-1 < 264 pg/mL, HR = 3.00, 95% CI = 1.37–6.98, *p* = 0.007; *BCR::ABL1* > 0.0036%, HR = 3.16, 95% CI = 1.12–7.69, *p* = 0.017; treatment duration < 111 months, HR = 2.38, 95% CI = 1.14–5.28, *p* = 0.025). The cumulative hazard analysis (Figure 6H) confirmed that low MCP-1, shorter treatment duration, and higher *BCR::ABL1* (%) levels were associated with a higher relapse risk.

## 4. Discussion

This study offers valuable insights into the variables potentially impacting molecular relapse-free survival after TKI discontinuation in CML patients, emphasizing clinical, molecular, and immune-related parameters.

Regarding risk stratification, while the Sokal score remains an important prognostic tool at diagnosis, its role in predicting post-discontinuation MRFS is less clear. In the STIM1 study, lower Sokal scores were associated with improved molecular remission rates [4]. However, findings from the EURO-SKI trial reported no significant association between Sokal or EUTOS scores and relapse rates following TKI discontinuation [15]. Although we found significant differences in Sokal scores between the NR and R groups, with a higher proportion of patients in the NR group showing low Sokal scores, this correlation does not seem to be a strong predictor for TFR. This suggests that, while the Sokal score remains useful for initial risk assessment, its capacity to predict successful TKI discontinuation outcomes is limited.

As expected, clinical and molecular variables such as treatment duration and depth of molecular response were associated with relapse outcomes, in line with previous studies. Factors such as total treatment duration and the depth of molecular response are critical determinants of sustained TFR. Our findings further reinforce the importance of the total duration of TKI therapy before discontinuation as a predictor of MRFS. Longer treatment duration has been associated with improved outcomes, as evidenced by studies such as the EURO-SKI trial, which reported a 63% MRFS at 6 months for patients treated for ≥5.8 years, compared to 41% for those treated for shorter durations [15]. Similarly, the STIM1 study found that patients with ≥50 months of imatinib therapy had a higher probability of sustained molecular remission and experienced significantly fewer molecular relapses than those with shorter treatment durations [4]. In our cohort, the treatment duration threshold associated with higher relapse risk was longer—111 months (approximately 9.25 years). Patients treated for less than this duration had a significantly increased relapse risk, and this effect remained consistent across bivariate and multivariate analyses. The Mann–Whitney test also confirmed significant differences in treatment duration between R and NR patients. Although our cutoff exceeds those reported in previous trials, this difference may reflect cohort-specific characteristics typical of real-world settings. Unlike clinical trials, our study likely included greater variability in treatment adherence and use of both treatment with brand or generic TKI formulations. While no significant difference in relapse risk was observed between original vs. copies TKIs, a longer treatment period may have been required to ensure a stable and sustained molecular response. These factors can influence the kinetics of molecular response and necessitate longer treatment periods before achieving a stable remission suitable for discontinuation.

Regarding molecular response, achieving and maintaining a DMR (MR4.5 or greater) at the time of cessation of TKI strongly predicts higher MRFS rates [16,17]. In our study, stratification by a *BCR::ABL1* (%) threshold of 0.0036%, close to the MR4.5 international standard (0.0032%), showed that patients below this threshold had significantly better outcomes. Moreover, detectable vs. undetectable *BCR::ABL1* transcripts at discontinuation were significantly associated with relapse risk, underscoring the importance of both depth and quality of molecular response. These results reinforce the value of sensitive and standardized molecular monitoring to refine eligibility for treatment discontinuation. Although DMR duration has been proposed as a prognostic factor for treatment-free remission, it did not reach significance in our cohort. This likely reflects how it was defined, from the first achievement of DMR until TKI discontinuation, without continuous confirmation of stability across the entire period. Since molecular monitoring tends to be more consistent in the years preceding discontinuation, this variable may underestimate the true persistence of deep response, which could explain its limited prognostic value in our analysis.

While our earlier analysis of the initial AST-I cohort suggested that low MCP-1 and IL-6 levels at discontinuation might be linked to relapse [12], the present study allows a more grounded evaluation of that signal. The extended follow-up of AST-I, together with the inclusion of the independent AST-II cohort, increased the number of informative events and gave us the opportunity to re-examine those associations with greater statistical confidence. AST-II also brings an important contextual difference: it was recruited after the COVID-19 pandemic, a period in which our group reported clear shifts in immune-cell subsets among TFR candidates [18]. Despite these changes in cellular immunity, the cytokine profiles remained remarkably consistent across both cohorts, and the relapse-associated MCP-1/IL-6 pattern persisted. This stability, even under different immunological backgrounds, strengthens the robustness of the cytokine signal and underscores its relevance in real-world settings.

In line with our previous observations, we also examined the MCP-1^low^/IL-6^low^ pattern as a single cytokine variable within the multivariable model. In the expanded dataset, this combined pattern remained significantly associated with relapse and showed a performance very similar to the model where MCP-1 and IL-6 were entered separately. This consistency across specifications reinforces the robustness of the cytokine signal in the context of TFR.

From an interpretative point of view, the two approaches highlight complementary aspects of the association. When the cytokines are included as individual covariates, MCP-1 retains an independent effect after adjustment, whereas IL-6 does not reach significance, suggesting that MCP-1 carries most of the predictive weight. When analyzed as a combined low-level pattern, the hazard ratio increases slightly, but the overall discrimination of the model remains essentially unchanged, as reflected by the similar Harrell’s C-index. Taken together, these results indicate that the underlying cytokine association is stable and does not depend on whether MCP-1 and IL-6 are considered separately or as a single low-level pattern. This stability strengthens the biological plausibility of the finding and supports the inclusion of cytokine-based markers alongside clinical variables when assessing relapse risk at discontinuation. These observations set the stage for one of the central contributions of this work, which is the integration of cytokine profiles into the prediction of molecular relapse, extending beyond traditional clinical and molecular markers. Our study suggests a potential role for IL-6 and MCP-1, in predicting molecular relapse following TKI discontinuation. These cytokines were key variables in a predictive decision tree model, which demonstrated strong performance in both the discovery and validation cohorts. The cytokine model achieved high specificity and a Positive Predictive Value of 100% in the validation cohort, indicating that when the model predicts relapse, it is highly reliable.

These findings support the notion that cytokine profiles may reflect aspects of the immune environment relevant to relapse risk. In our previous work [12], we proposed that IL-6 and MCP-1 could influence leukemic stem cell (LSC) dynamics in the setting of long-term TKI exposure. In that framework, persistent IL-6 mediated proliferative pressure on LSCs was hypothesized to promote their functional exhaustion, while MCP-1 may exert a differential effect on hematopoietic stem cells, potentially contributing to the preservation of normal hematopoiesis. Although these mechanisms were not directly addressed here, they provide a useful basis to interpret the cytokine patterns observed in this study. In the present study, our data extend this perspective by suggesting that this cytokine profile may also reflect a treatment-associated state of immune vigilance that is progressively established during TKI therapy and persists at the time of treatment discontinuation. Under prolonged TKI exposure, patients may develop a stabilized equilibrium in which residual *BCR::ABL1*-positive cells are maintained under continuous control. Within this context, IL-6 and MCP-1 do not simply act as markers of inflammation, but rather as components of a dynamic environment that integrates leukemic cell-intrinsic pressure with immune-mediated surveillance. Interestingly, when our data are considered in light of previously reported cytokine levels in healthy donors measured using the same methodology [19], median concentrations of IL-6 and MCP-1 were reported as 0 and 155.9 pg/mL, respectively (*n* = 19). In this context, the observed thresholds in our cohort do not correspond to a return to physiological baseline. Instead, they suggest that relapse may be associated with cytokine levels dropping below a range that remains compatible with effective immune surveillance. Notably, both relapsed and non-relapsed patients exhibit cytokine levels above those typically observed in healthy individuals, consistent with a treatment-adapted state rather than a return to baseline physiology. In this context, IL-6 and MCP-1 are better interpreted as indicators of a sustained, vigilance-associated immune environment rather than simple markers of inflammation. While both cytokines are typically elevated in CML and associated with poor prognosis [20,21,22], we observed that lower levels were linked to a higher risk of relapse. Sustained elevated levels of these cytokines may correspond to a microenvironment that facilitates the recruitment and activation of NK cells, CD8+ cytotoxic T cells, and activated monocytes/macrophages at niches where residual *BCR::ABL1* cells persist. This model does not dismiss the inflammatory role of IL-6 and MCP-1; rather, it positions these cytokines as functional biomarkers of immune surveillance capacity, consistent with previous evidence describing their dual and context-dependent roles in tumor immunity and hematologic malignancies [23,24,25].

Survival and hazard analyses further highlight the importance of cytokines as prognostic factors, when combined with elevated *BCR::ABL1* levels and shorter treatment durations. Patients in the MCP-1^low^/IL-6^low^, *BCR::ABL1* > 0.0036%, and treatment duration < 111 months experienced the sharpest decline in molecular relapse-free survival, with a significant rise in cumulative hazard, suggesting a combined influence of immune dysregulation and residual molecular disease.

Among all combinations tested, the model integrating MCP-1, *BCR::ABL1* (%), and treatment duration achieved the highest concordance according to Harrell’s index, indicating superior prognostic discrimination. This pattern likely reflects the real-world characteristics of our cohort, where prolonged TKI exposure was common. Treatment duration may thus capture not only therapy length but also the cumulative biological stability achieved through sustained molecular response, reinforcing its joint role with cytokine and molecular parameters in relapse prediction. Clinically, this integrated approach could guide individualized monitoring intensity or support delaying TKI cessation in high-risk patients until a more favorable immune-molecular balance is achieved.

The decision tree and the Cox proportional hazards model address relapse from different methodological perspectives and their results are complementary. Together, these results support the integration of cytokine biomarkers into existing prognostic models, offering an additional layer of biologically grounded risk assessment that may inform more precise and individualized therapeutic strategies in the context of treatment-free remission in CML.

Beyond their statistical performance, these findings open the possibility of integrating cytokine profiling into current molecular monitoring strategies for CML. Given that *BCR::ABL1* transcript quantification by RT-qPCR is already established as the standard tool for treatment decision-making, cytokine assessment could serve as a complementary layer of prognostic information at the time of TKI discontinuation, particularly for patients with borderline molecular results. With appropriate validation, this integration could be implemented through accessible methodologies such as ELISA or emerging qPCR-based immunoassays, allowing cytokine measurement within the same analytical framework already used for *BCR::ABL1* monitoring. Such an approach could facilitate the translation of cytokine profiling into clinical practice, bridging molecular and immunological markers to refine patient selection for treatment-free remission.

Given these findings, could the combination of cytokine profiling, molecular response depth and time of treatment be translated into clinical decision-making to refine eligibility for TKI discontinuation in chronic phase CML? If so, how might such information reshape post-discontinuation monitoring strategies or thresholds for intervention? Patients identified as high-risk based on these profiles may not be the best candidates for immediate TKI discontinuation. For these individuals, an extension in treatment duration or exploration of novel immunomodulatory strategies could help to optimize TFR decision. In addition, low-risk patients, particularly those with MCP-1^high^ profiles and favorable molecular markers, may be better suited for TKI cessation, given their lower relapse risk. The stratification observed in our cohort based on MCP-1 and IL-6 levels raises the possibility of adapting molecular follow-up intensity according to individual immunological risk profiles. On the other hand, patients falling within the subgroup defined by low MCP-1 and IL-6 concentrations might benefit from more frequent molecular assessments or extended TKI therapy prior to cessation. These considerations reflect an evolving understanding of TFR not only as a function of molecular depth and treatment duration, but also of immune competence. Incorporating such markers into prospective clinical frameworks may help identify patients who, despite meeting conventional molecular criteria, remain at elevated risk of relapse due to insufficient immune control.

## 5. Conclusions

Cytokine profiling adds important prognostic value and enriches the decision-making process for TKI discontinuation. At the same time, its broader application in clinical practice may depend on further efforts to standardize and harmonize multiplex cytokine measurement methods, improving their translatability. While our findings are based on statistical modeling and do not establish direct causality, they raise important hypotheses about the immune microenvironment supporting sustained TFR. Cytokine alterations may reflect early immune imbalances preceding relapse, offering a nuanced and biologically insightful approach to assessing TFR sustainability. Future mechanistic studies will be essential to clarify the functional roles of these cytokines. Expanding sample sizes, especially in validation cohorts, will also help strengthen the predictive power of these biomarkers. Overall, our findings suggest that cytokines such as IL-6 and MCP-1 may play an active role in CML immunobiology and could contribute to the early identification of patients at higher risk of relapse, thus informing more personalized discontinuation strategies.

## Figures and Tables

**Table 1:** Characteristics of patients at baseline.

Characteristics	Reference	Relapsed (*n* = 32)	Non-Relapsed (*n* = 49)	R vs. NR (*p*-Value)
Age (years) at diagnosis: median (range)		45 (16–73)	44 (21–71)	0.703
Age (years) at enrollment: median (range)		52 (25–80)	58 (29–86)	0.569
Sex	Female	17	27	0.861
	Male	15	22	
Treatment duration: median (range)		88 (52–218)	132 (50–263)	0.013 *
Treatment duration (months)	<111	22	20	0.014 *
	>111	10	29	
*BCR::ABL1* detectability at enrollment	Detectable	18	16	0.035
	Undetectable	14	33	
Duration of DMR until Discontinuation (months): median (range)		61 (30–179)	80 (34–203)	0.030 *
Time to First DMR (from Treatment Start–months): median (range)		23 (2–139)	27 (3–138)	0.147
Time to First DMR (from Treatment Start)	<27 months	21	11	0.195
	>27 months	25	24	
TKI type prior to stop	Imatinib	25	40	0.698
	2GTKI	7	9	
*BCR::ABL1* (%): median (range)		0.0009 (0–0.0082)	0.0014 (0–0.0055)	0.028 *
*BCR::ABL1* (%)	<0.0036%	25	49	0.007 *
	>0.0036%	6	1	
Sokal score	Low	12	28	0.044 *
	High/Inter	19	17	

Abbreviations: R, Relapsed; NR, Non-relapsed; DMR, Deep Molecular Response; TKI, Tyrosine-Kinase Inhibitor; 2GTKI, second-generation TKI. * Statistically significant *p*-value.

**Table 2:** Cytokine Levels in Plasma Samples (AST-I Cohort).

Analyte	Non-Relapsed (*n* = 27) Mean (Range)	Relapsed (*n* = 19) Mean (Range)	R vs. NR (*p*-Value)
CD86	24,032 (7206–53,805)	23,123 (6751–54,051)	0.882
Eotaxin	182.3 (65.6–379.3)	142.4 (63.8–245.3)	0.114
G-CSF	106.4 (52.6–190.3)	117.7 (44.4–256.4)	0.770
GM-CSF	50.7 (8.6–146.2)	81 (11.9–165.1)	0.075
IFNa2	108.5 (67.4–153.5)	104.7 (54.2–153.4)	0.850
IL-10	4.4 (0.4–14.7)	3.2 (0.4–7)	0.558
IL-12(p70)	2.9 (0.5–8.2)	6 (0.9–10.9)	0.007 *
IL-15	16.4 (11.2–23.6)	16.3 (10.4–20.9)	0.895
IL-1a	42.9 (23.4–75.1)	36.6 (19.5–63.6)	0.209
IL-1b	19.5 (11.3–34.3)	18.8 (7.9–32.1)	0.670
IL-1RA	7.5 (4.2–13.8)	7.3 (3.7–13.8)	0.977
IL-2	2.6 (1.8–4.5)	2.5 (2–3.6)	0.723
IL-4	3.7 (2.2–6)	3.7 (2.2–7.8)	>0.999
IL-6	3.8 (1.9–5.3)	3.2 (1.7–5.7)	0.015 *
IL-7	5 (2.5–9.5)	5.4 (2.7–10.9)	0.583
IL-8	3.3 (1.8–5.4)	2.8 (1.9–4.8)	0.029 *
IL-9	11.4 (5.2–21.2)	11.6 (7.8–14.5)	0.547
MCP-1	295.4 (198.8–427.4)	247.6 (147.1–460)	0.005 *
MIP-1a	13.8 (3.6–40.9)	18.9 (2–36.2)	0.098
PD-1	3364 (1506–6327)	3927 (1622–8758)	0.413
PD-L1	1705 (713–3931)	1842 (814–4223)	0.774
PD-L2	25,172 (8372–40,362)	23,054 (11,883–35,127)	0.214
SCF	86.2 (34.4–190.4)	81.4 (27–153.3)	0.909
TGF-a	5.2 (3.1–7.3)	4.9 (3.3–5.9)	0.686
TGF-b1	17,019 (2680–59,859)	25,607 (3831–63,755)	0.152
TGF-b2	962 (473.5–2009)	963 (460–2206)	0.641
TNF-a	18.7 (5.5–50.8)	21 (6.8–56.7)	0.882
VEGFA	61.2 (10.3–281.1)	99.1 (16.6–381.4)	0.130

Abbreviations: R, Relapsed; NR, Non-relapsed; CD, Cluster of Differentiation; G-CSF, granulocyte colony-stimulating factor; GM-CSF, Granulocyte-Macrophage Colony-Stimulating Factor; IFN, Interferon; IL, Interleukin; MCP, Monocyte chemoattractant protein; MIP, Macrophage Inflammatory Proteins; PD, Programmed Cell Death; SCF, Stem Cell Factor; TGF, Transforming Growth Factor; TNF, Tumor Necrosis Factor; VEGF, Vascular Endothelial Growth Factor. * Statistically significant *p*-value.

**Table 3:** Cytokine Levels in Plasma Samples (AST-I and AST-II Cohort).

Analyte	Non-Relapsed (*n* = 49) Mean (Range)	Relapsed (*n* = 32) Mean (Range)	R vs. NR (*p*-Value)
G-CSF	114.7 (7.6–222)	111.7 (44.4–256.4)	0.430
IL-10	5.8 (0.4–24.3)	5.5 (0.4–19)	0.505
IL-12(p70)	3.6 (0.5–10.6)	4.3 (0.5–10.9)	0.632
IL-6	3.9 (0.1–8.1)	2.9 (0.7–8.1)	0.007 *
MCP-1	289.1 (166.1–427.4)	253.5 (147.1–460.1)	0.012 *
MIP-1a	18.8 (3.6–41)	26.3 (2–77.1)	0.229
TNF-a	19.8 (3.7–50.8)	20.2 (2.9–56.7)	0.610
VEGFA	67.8 (10.6–222.3)	50.2 (14.3–141.4)	0.706

Abbreviations: R, Relapsed; NR, Non-relapsed; G-CSF, granulocyte colony-stimulating factor; IL, Interleukin; MCP, Monocyte chemoattractant protein; MIP, Macrophage Inflammatory Proteins; TNF, Tumor Necrosis Factor; VEGF, Vascular Endothelial Growth Factor. * Statistically significant *p*-value.

**Table 4:** Predictive Performance of Clinical Variables and Cytokines in Discovery and Validation Cohorts.

	Clinical Model		Cytokines Model	
	Discovery Cohort	Validation Cohort	Discovery Cohort	Validation Cohort
AUC	0.7063	0.6969	0.7643	0.7708
Accuracy	0.7000	0.7000	0.7667	0.8000
Sensitivity	0.6818	0.7778	0.6957	0.5000
Specificity	0.7105	0.6364	0.8108	1.000
PPV	0.5769	0.6364	0.6957	1.000
NPV	0.7941	0.7778	0.8108	0.7500
*p*-value	0.1746	0.1299	0.0102	0.0509

Abbreviations: AUC, Area Under the Curve; PPV, Positive Predictive Value; NPV, Negative Predictive Value.

**Table 5:** Performance of Cytokines model in Relapse Prediction: Confusion Matrix Analysis for Discovery and Validation Cohorts.

	**Discovery Cohort Prediction**	
	**Observed Non-Relapsed**	**Observed Relapsed**
**Predicted to** **Non-relapsed (*n* = 37)**	30 True negative 50%	7 False negative 11.67%
**Predicted to** **Relapsed (*n* = 23)**	7 False positive 11.67%	16 True positive 26.67%
	**Validation cohort prediction**	
	**Observed Non-relapsed**	**Observed Relapsed**
**Predicted to** **Non-relapsed (*n* = 16)**	12 True negative 60%	4 False negative 20%
**Predicted to** **Relapsed (*n* = 4)**	0 False positive 0%	4 True positive 20%

**Table 6:** Univariable Cox regression analysis, with hazard ratios for molecular recurrence throughout the entire follow end up period.

Variable	Reference	HR (95% CI)	*p*-Value	Harrell’s C (95% CI)
MCP-1	<264 pg/mL	3.45 (1.64–7.80)	0.002 *	0.64 (0.55–0.73)
IL-6	<4.5 pg/mL	3.13 (1.11–13.11)	0.060	0.58 (0.51–0.65)
IL-3		1.32 (0.92–1.84)	0.112	0.56 (0.47–0.66)
IL-10		0.95 (0.86–0.99)	0.160	0.61 (0.50–0.72)
MIP-1a		1.01 (0.98–1.03)	0.240	0.53 (0.42–0.64)
TNFa		1.01 (0.99–1.02)	0.306	0.51 (0.40–0.62)
G-CSF		1.00 (0.99–1)	0.627	0.42 (0.30–0.54)
IL-12		1.00 (0.97–1)	0.715	0.50 (0.38–0.62)
VEGFA		1.00 (0.99–1)	0.825	0.51 (0.40–0.62)
*BCR::ABL1* (%)	>0.0036%	4.23 (1.54–9.92)	0.002 *	0.57 (0.51–0.64)
Treatment duration	<111 months	2.36 (1.13–5.23)	0.026 *	0.60 (0.50–0.69)
*BCR::ABL1* detectability at enrollment	Detectable	2.19 (1.07–4.52)	0.031 *	0.60 (0.51–0.69)
Sokal score	High/Inter	1.90 (0.92–4.06)	0.084	0.58 (0.48–0.68)
Duration of DMR until discontinuation		0.99 (0.97–1)	0.089	0.59 (0.49–0.69)
Time to First DMR	<27 months	1.66 (0.80–3.58)	0.180	0.56 (0.47–0.65)
DMR at discontinuation	MR4.0	1.88 (0.64–5.10)	0.224	0.57 (0.47–0.67)
TKI type prior to stop	2GTKI	1.30 (0.52–2.88)	0.537	0.53 (0.45–0.62)
Age at diagnosis		0.99 (0.96–1.02)	0.728	0.50 (0.38–0.61)
Sex	Male	1.03 (0.49–2.08)	0.944	0.51 (0.41–0.60)

Abbreviations: HR, Hazard Ratios; CI, Confidence Interval; MCP, Monocyte chemoattractant protein; IL, Interleukin; MIP, Macrophage Inflammatory Proteins; TNF, Tumor Necrosis Factor; G-CSF, granulocyte colony-stimulating factor; VEGF, Vascular Endothelial Growth Factor; DMR, Deep Molecular Response; TKI, Tyrosine-Kinase Inhibitor; 2GTKI, second-generation TKI. * Statistically significant *p*-value.

**Table 7:** Multivariable Cox regression analysis, with hazard ratios for molecular recurrence throughout the entire follow-up period.

Variable	Reference	HR (95% CI)	*p*-Value	Harrell’s C (95% CI)
MCP-1	<264 pg/mL	2.94 (1.28–7.11)	0.012 *	0.77 (0.69–0.85)
IL-6	<4.5 pg/mL	1.92 (0.59–8.66)	0.324	
IL-10		0.96 (0.86–1)	0.331	
G-CSF		1.00 (0.99–1)	0.654	
IL-12		1.00 (0.96–1.01)	0.979	
VEGFA		1.00 (0.99–1)	0.989	
*BCR::ABL1* (%)	>0.0036%	3.62 (1.01–12.65)	0.042 *	
Time to First DMR	<27 months	2.18 (0.98–5.05)	0.059	
Sex	Male	2.12 (0.88–5.37)	0.098	
Age at diagnosis		1.02 (0.98–1.06)	0.244	
Sokal Score	High/Inter	1.53 (0.66–3.59)	0.316	
*BCR::ABL1* detectability at enrollment	Detectable	1.55 (0.61–3.83)	0.343	
TKI type prior to stop	2GTKI	0.83 (0.31–2)	0.702	

Abbreviations: HR, Hazard Ratios; CI, confidence Interval; MCP, Monocyte chemoattractant protein; IL, Interleukin; G-CSF, granulocyte colony-stimulating factor; VEGF, Vascular Endothelial Growth Factor; DMR, Deep Molecular Response; TKI, Tyrosine-Kinase Inhibitor; 2GTKI, second-generation TKI. * Statistically significant *p*-value.

**Table 8:** Multivariable Cox regression analysis including the MCP-1^low^/IL-6^low^ combined cytokine pattern and clinical variables, with hazard ratios (HR) for molecular recurrence throughout the entire follow-up period.

Variable	Reference	HR (95% CI)	*p*-Value	Harrell’s C (95% CI)
MCP-1 + IL-6	<264 pg/mL:<4.5 pg/mL	3.47 (1.55–8.08)	0.003 *	0.77 (0.68–0.85)
*BCR::ABL1*	>0.0036%	3.43 (0.96–11.96)	0.051	
Time to First DMR	<27 months	2.21 (0.99–5.19)	0.057	
Sex	Male	2.16 (0.89–5.54)	0.092	
Age at diagnosis		1.02 (0.98–1.06)	0.251	
Sokal Score	High/Inter	1.50 (0.64–3.54)	0.342	
IL-10		0.95 (0.85–1)	0.303	
*BCR::ABL1* detectability at enrollment	Detectable	1.62 (0.62–4.05)	0.301	
G-CSF		1.00 (0.99–1)	0.667	
TKI type prior to stop	2GTKI	0.83 (0.31–1.97)	0.687	
IL-12		1.00 (0.96–1.01)	0.885	
VEGFA		1.00 (0.99–1)	0.998	

Abbreviations: HR, Hazard Ratios; CI, confidence Interval; MCP, Monocyte chemoattractant protein; IL, Interleukin; G-CSF, granulocyte colony-stimulating factor; VEGF, Vascular Endothelial Growth Factor; DMR, Deep Molecular Response; TKI, Tyrosine-Kinase Inhibitor; 2GTKI, second-generation TKI. * Statistically significant *p*-value.

**Table 9:** Bivariate and trivariate Cox regression analysis, with hazard ratios for molecular recurrence throughout the entire follow-up period.

Variable	Reference	HR (95% CI)	*p*-Value	Harrell’s C (95% CI)
Model (1)				
MCP-1	<264 pg/mL	3.54 (1.68–8.00)	0.001 *	0.68 (0.59–0.77)
IL-6	<4.5 pg/mL	3.28 (1.16–13.75)	0.050	
Model (2)				
MCP-1	<264 pg/mL	3.06 (1.42–7.01)	0.006 *	0.68 (0.59–0.77)
*BCR::ABL1* (%)	>0.0036%	3.10 (1.15–7.46)	0.018 *	
Model (3)				
MCP-1	<264 pg/mL	3.48 (1.64–7.92)	0.002 *	0.69 (0.59–0.78)
Treatment duration	<111 months	2.36 (1.13–5.25)	0.026 *	
Model (4)				
*BCR::ABL1* (%)	>0.0036%	4.59 (1.67–10.86)	0.001 *	0.64 (0.55–0.75)
Treatment duration	<111 months	2.49 (1.19–5.53)	0.018 *	
Model (5)				
MCP-1	<264 pg/mL	3.00 (1.37–6.98)	0.007 *	0.72 (0.62–0.81)
*BCR::ABL1* (%)	>0.0036%	3.16 (1.12–7.69)	0.017 *	
Treatment duration	<111 months	2.38 (1.14–5.28)	0.025 *	

Abbreviations: HR, Hazard Ratios; CI, confidence Interval; MCP, Monocyte chemoattractant protein; IL, Interleukin. * Statistically significant *p*-value.

## Data Availability

The data presented in this study are available on request from the corresponding author. The data are not publicly available due to privacy and ethical restrictions related to patient information.

## Supplementary Materials

The following supporting information can be downloaded at: https://www.mdpi.com/article/10.3390/cells15090791/s1, Table S1: Inclusion and exclusion criteria for patient enrollment in the Argentina Stop Trial—AST.; Table S2: Baseline clinical characteristics of patients from AST-I and AST-II cohorts.

## Abbreviations

The following abbreviations are used in this manuscript:

AST	Argentina Stop Trial
AUC	Area Under the Curve
CD	Cluster of Differentiation
CI	Confidence Interval
CML	Chronic Myeloid Leukemia
DMR	Deep Molecular Response
G-CSF	Granulocyte Colony-Stimulating Factor
GM-CSF	Granulocyte-Macrophage Colony-Stimulating Factor
HR	Hazard Ratios
IFN	Interferon
IL	Interleukin
MCP	Monocyte Chemoattractant protein-1
MIP	Macrophage Inflammatory Proteins
MMR	Major Molecular Response
MR	Molecular Response
MRFS	Molecular Relapse-Free Survival
NK	Natural Killer
NR	Non-Relapsed
PD	Programmed Cell Death
R	Relapsed
ROC	Receiver Operating Characteristic
SCF	Stem Cell Factor
TFR	Treatment Free Remission
TGF	Transforming Growth Factor
TKI	Tyrosine Kinase Inhibitor
2GTKI	Second-Generation TKI
TNF	Tumor Necrosis Factor
VEGF	Vascular Endothelial Growth Factor
