# Supplementary material for: Identification of Predictive Biomarkers Based on Cytokine Profiles for Molecular Relapse After Treatment-Free Remission in Chronic Myeloid Leukemia Patients
Source: Cells. 2026 Apr 27;15(9):791. doi: 10.3390/cells15090791 (PMC13162600; doi:10.3390/cells15090791)
Supplement: Supplementary file 1 [file cells-15-00791-s001.zip › cells-4226413-supplementary.pdf]

Table S1. Inclusion and exclusion criteria for patient enrollment in the Argentina Stop Trial – AST.

Category	Criteria
Inclusion Criteria	• Adult patients (≥ 18 years) with diagnosis CP-CML, *BCR::ABL1*+<br>• Treatment with TKI (Imatinib, Nilotinib, or Dasatinib; original or generic formulations), in first line or second line due to intolerance<br>• Classical *BCR::ABL1* transcript isoforms (b3a2 [e14a2] and/or b2a2 [e13a2])<br>• Achievement of DMR (≥ MR4.0; *BCR::ABL1* ≤ 0.01% or undetectable *BCR::ABL1* with ≥ 10,000 ABL1 transcripts), assessed in laboratories standardized to the International Scale<br>• Sustained DMR (≥MR4.0) for at least 2 years, documented by ≥ 4 molecular assessments performed at intervals ≥ 3 months<br>• Minimum duration of TKI treatment ≥ 54 months prior to discontinuation
Exclusion Criteria	• History of treatment failure to any TKI at any time<br>• Progression to accelerated phase or blast crisis at any time<br>• Presence of TKI resistance-associated mutations

Abbreviations: CP-CML, Chronic Phase - Chronic Myeloid Leukemia; DMR, Deep Molecular Response; TKI, Tyrosine-Kinase Inhibitor; MR, Molecular Response.

Table S2. Baseline clinical characteristics of patients from AST-I and AST-II cohorts.

Characteristics	Reference	AST-I (n = 46)	AST-II (n = 35)	AST-I vs AST-II (p-value)
Age (years) at diagnosis: median (range)		45 (16 – 73)	43 (21 – 65)	0.262
Age (years) at enrollment: median (range)		58 (25 - 86)	52 (29 – 75)	0.074
Sex	Female	24	20	0.822
	Male	22	15	
Treatment duration: median (range)		122 (52 – 227)	98 (50 – 263)	0.410
BCR::ABL1 detectability at enrollment	Detectable	18	16	0.650
	Undetectable	28	19	
Duration of DMR until Discontinuation (months): median (range)		77 (30 – 160)	74 (35 – 203)	0.587
Time to First DMR (from Treatment Start - months): median (range)		31 (2 – 139)	27 (3 – 129)	0.254
TKI type prior to stop	Imatinib	37	28	>0.999
	2GTKI	9	7	
BCR::ABL1 (%): median (range)	2422	0.0000 (0 - 0.0082)	0.0000 (0 – 0.0077)	0.643
Sokal score	Low	22	18	0.351
	High/Inter	24	12	

Abbreviations: R, Relapsed; NR, Non-relapsed; DMR, Deep Molecular Response; TKI, Tyrosine-Kinase Inhibitor; 2GTKI, second-generation TKI. * Statistically significant p-value.
